# Flexibilide Obtained from Cultured Soft Coral Has Anti-Neuroinflammatory and Analgesic Effects through the Upregulation of Spinal Transforming Growth Factor-β1 in Neuropathic Rats

**DOI:** 10.3390/md12073792

**Published:** 2014-06-27

**Authors:** Nan-Fu Chen, Shi-Ying Huang, Ching-Hsiang Lu, Chun-Lin Chen, Chien-Wei Feng, Chun-Hong Chen, Han-Chun Hung, Yen-You Lin, Ping-Jyun Sung, Chun-Sung Sung, San-Nan Yang, Hui-Min David Wang, Yu-Chia Chang, Jyh-Horng Sheu, Wu-Fu Chen, Zhi-Hong Wen

**Affiliations:** 1 Department of Marine Biotechnology and Resources, Asia-Pacific Ocean Research Center, National Sun Yat-sen University, Kaohsiung 80424, Taiwan; E-Mails: chen06688@gmail.com (N.-F.C.); johnjohnkings@gmail.com (S.-Y.H.); qscjuejuejue@gmail.com (C.-W.F.); anubis0620@gmail.com (C.-H.C.); hanchun25@gmail.com (H.-C.H.); chas6119@gmail.com (Y.-Y.L.); sheu@mail.nsysu.edu.tw (J.-H.S.); 2 Division of Neurosurgery, Department of Surgery, Kaohsiung Armed Forces General Hospital, Kaohsiung 80284, Taiwan; E-Mails: alfaleu@gmail.com (C.-H.L.); springwei@hotmail.com.tw (C.-L.C.); 3 Department and Graduate Institute of Aquaculture, National Kaohsiung Marine University, Kaohsiung 81157, Taiwan; 4 Doctoral Degree Program in Marine Biotechnology, National Sun Yat-sen University and Academia Sinica, Kaohsiung 80424, Taiwan; E-Mail: jay0404@gmail.com; 5 National Museum of Marine Biology and Aquarium, Pingtung 944, Taiwan; E-Mail: pjsung@nmmba.gov.tw; 6 Department of Anesthesiology, Taipei Veterans General Hospital, Taipei 11217, Taiwan; E-Mail: cssung@vghtpe.gov.tw; 7 School of Medicine, National Yang-Ming University, Taipei 11221, Taiwan; 8 School of Medicine, College of Medicine and Department of Pediatrics, E-DA Hospital, I-Shou University, Kaohsiung 84001, Taiwan; E-Mail: y520729@gmail.com; 9 Department of Fragrance and Cosmetic Science, Kaohsiung Medical University, Kaohsiung 80708, Taiwan; E-Mail: davidw@kmu.edu.tw; 10 Graduate Institute of Natural Products, Kaohsiung Medical University, Kaohsiung 80708, Taiwan; 11 Department of Neurosurgery, Kaohsiung Chang Gung Memorial Hospital and Chang Gung University College of Medicine, Kaohsiung 83301, Taiwan

**Keywords:** flexibilide, chronic constriction injury, neuropathic pain, spinal neuroinflammation, microglial activation, transforming growth factor-β1, natural marine compound

## Abstract

Chronic neuroinflammation plays an important role in the development and maintenance of neuropathic pain. The compound flexibilide, which can be obtained from cultured soft coral, possesses anti-inflammatory and analgesic effects in the rat carrageenan peripheral inflammation model. In the present study, we investigated the antinociceptive properties of flexibilide in the rat chronic constriction injury (CCI) model of neuropathic pain. First, we found that a single intrathecal (i.t.) administration of flexibilide significantly attenuated CCI-induced thermal hyperalgesia at 14 days after surgery. Second, i.t. administration of 10-μg flexibilide twice daily was able to prevent the development of thermal hyperalgesia and weight-bearing deficits in CCI rats. Third, i.t. flexibilide significantly inhibited CCI-induced activation of microglia and astrocytes, as well as the upregulated proinflammatory enzyme, inducible nitric oxide synthase, in the ipsilateral spinal dorsal horn. Furthermore, flexibilide attenuated the CCI-induced downregulation of spinal transforming growth factor-β1 (TGF-β1) at 14 days after surgery. Finally, i.t. SB431542, a selective inhibitor of TGF-β type I receptor, blocked the analgesic effects of flexibilide in CCI rats. Our results suggest that flexibilide may serve as a therapeutic agent for neuropathic pain. In addition, spinal TGF-β1 may be involved in the anti-neuroinflammatory and analgesic effects of flexibilide.

## 1. Introduction

Various natural marine organisms can be promising sources for medicinal substances that can be used against various infections and diseases such as diabetes mellitus and cancer [1,2,3]. Some marine compounds also have anti-inflammatory effects [4,5,6]. Therefore, it is likely that marine-derived compounds have great potential for drug development in many diseases currently considered difficult to treat [7,8,9,10].

Neuropathic pain remains one of most challenging diseases for clinicians, with current treatments considered unsatisfactory for many patients [11]. Inflammatory processes are involved in both the peripheral and the central nervous system (CNS) and are thought to be involved in the pathogenesis of neuropathic pain [12,13]. The activation of astrocytes and microglia cells plays critical roles in neuroinflammatory processes [14,15]. It has also been extensively described that activated astrocytes and microglial cells can increase synthesis and release of proinflammatory cytokines, such as interleukin-1β (IL-1β), interleukin-6 (IL-6), tumor necrosis factor-α (TNF-α), prostaglandin E2 (PGE2), and nitric oxide (NO) [16,17]. These mediators can further enhance neuroinflammation and thereby lead to the sensitization of nociceptive transmission [18]. In addition, activated microglia and astrocytes are implicated in the initiation and maintenance of spinal nociceptive sensitization in neuropathic pain states [19,20,21]. Inhibition of glia (microglia and astrocytes) activation has been recently proposed as a novel way of controlling neuropathic pain [20,22,23]. Therefore, analgesic efficacy of many novel compounds may depend on their ability to inhibit microglia and astrocyte activation [24,25,26,27].

Peripheral nerve injury is associated with local inflammatory processes at the site of injury. For example, nerve injury can initiate local inducible nitric oxide synthase (iNOS) expression in both macrophages and Schwann cells within and distal to the injury site [28]. A dramatic increase in the number of NOS mRNA-positive neurons in the L4 and L5 ganglia has been reported after peripheral axotomy [29]. Many studies have reported that NO is involved in acute pain as well as in the development and maintenance of neuropathic pain [30,31]. Intrathecal (i.t.) administration of nitroglycerin, an NO donor, produces thermal hyperalgesia in rats [32]. Inhibition of spinal NO synthesis also reduces neuropathic pain [31,33]. Therefore, central activation of the iNOS-NO system is likely involved in the pathogenesis of neuropathic pain.

The proteins in the transforming growth factor-β (TGF-β) family are disulphide-linked multifunctional polypeptides, which have been implicated in a broad range of biological functions including inhibition and stimulation of cell proliferation, immunosuppression, chemoprotection, tissue repair, and neuroprotection [34,35,36,37]. Systemic administration of TGF-β1 greatly alleviates the inflammatory response in streptococcalcell wall-induced erosive arthritis and prevents the relapse of autoimmune encephalomyelitis [38,39]. TGF-β1 has also been reported to inhibit proliferation of microglia and astrocytes both *in vitro* and *in vivo* [40,41,42,43]. We have previously demonstrated that i.t. TGF-β1 attenuates thermal hyperalgesia and spinal microglial and astrocytic activation in chronic constriction injury (CCI) rats [44].

**Figure 1:**
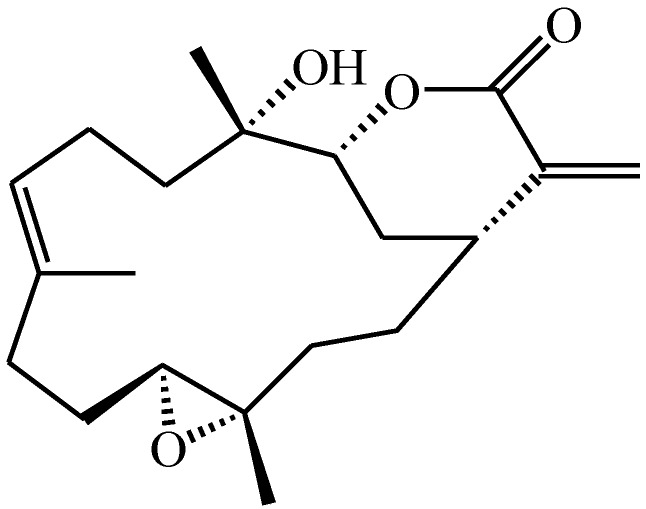
Chemical structure of 5,15-dioxatricyclo[12.3.1.0(4,6)]octadec-9-en-16-one (flexibilide).

The natural marine compound flexibilide (Figure 1) was originally isolated from the soft coral *Sinularia flexibilis* from the Hayman Island located on the Great Barrier Reef of Australia [45]. The structure of this compound was first reported by Weinheimer’s group in 1977 and given the name sinularin [45]. Shortly afterwards, it was reported by the Roche group in Sydney [46], but given the name flexibilide. Both groups established the structure by X-ray crystallography. The original name, sinularin, has rarely been used in subsequent publications referring to this molecule, and the name flexibilide has been used far more commonly. Anti-inflammatory effects for flexibilide were first reported by Buckle *et al.* [47] who showed that it could reduce carrageenan-induced paw edema and inhibit adjuvant-induced paw swelling. Flexibilide can also significantly inhibit upregulation of iNOS and cyclooxygenase-2 (COX-2) in lipopolysaccharide (LPS)-stimulated murine macrophage RAW 264.7 cells. In addition to its anti-inflammatory effects, flexibilide also exerts antinociceptive effects in carrageenan-induced nociceptive pain [6]. However, the antinociceptive mechanisms of flexibilide and its potential use in neuropathic pain remain unclear.

In the present study, we investigate the antinociceptive effects of i.t. flexibilide on the well-established CCI rat model of neuropathic pain. In order to examine potential CNS mechanisms involved, we also measure changes in iNOS, TGF-β1, and glial cell activation in the dorsal horn (DH) of the spinal cord in CCI rats treated with flexibilide.

## 2. Results

### 2.1. Flexibilide Attenuates the CCI-Induced Thermal Hyperalgesia

**Figure 2:**
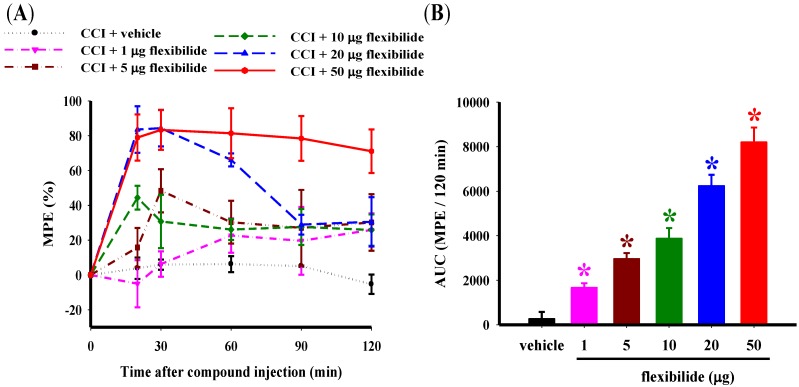
Effect of i.t. vehicle or flexibilide on thermal hyperalgesia in rats with CCI. (**A**) Time course for the PWL for vehicle and various doses of i.t. flexibilide in CCI rats. The Y-axis shows the %MPE calculated as the mean with each dose and the X-axis shows thetime in minutes from the time of the i.t. injection of flexibilide or vehicle; (**B**) The area under the curve (%MPE-time curve as mean ± SEM) for the i.t. vehicle and dose (1, 5, 10, 20, and 50 μg) of flexibilide. I.t. flexibilide has a dose-dependent effect on thermal hyperalgesia in CCI rats. *n* = 6 per group, * *p* < 0.05 as compared with the CCI + vehicle group. CCI: chronic constriction injury, PWL: paw withdrawal latency, MPE: maximum possible effect.

There were no significant differences among the experimental groups in the baseline paw withdrawal latency (PWL) before CCI surgery or sham surgery. The average PWL baseline was 28.51 ± 0.35 s (*n* = 36). As expected, thermal hyperalgesia (PWL = 13.01 ± 0.60 s) was observed in the ipsilateral hindpaw 14 days after CCI. Figure 2A shows the time course of the percentage maximum possible effect (%MPE) for reduction of thermal hyperalgesia with i.t. flexibilide at doses of 1, 5, 10, 20, and 50 μg. Figure 2B presents the duration of this antinociceptive effect as shown by the area under the curve, which ranges from 0 to 120 min after the i.t. flexibilide injection. Rapid antinociception was achieved as early as 20 min after injection. Both vehicle and flexibilide injections did not alter the PWL in naïve rats. I.t. injection of vehicle (2% dimethylsulfoxide, DMSO) also did not affect CCI-induced thermal hyperalgesia. As compared to the vehicle group, i.t. flexibilide produced a significant dose-dependent antinociceptive effect in neuropathic rats. The Basso, Beattie, and Bresnahan (BBB) rating scale was used to evaluate potential motor effects of the i.t. flexibilide. Sham-operated rats were treated with i.t. flexibilide at doses of 10, 20, and 50 μg and it indicated normal locomotor function (BBB score = 25 and *n* = 6 for each group).

### 2.2. Prophylactic i.t. Flexibilide Prevents the Development of CCI-Induced Thermal Hyperalgesia and Weight-Bearing Deficits

**Figure 3:**
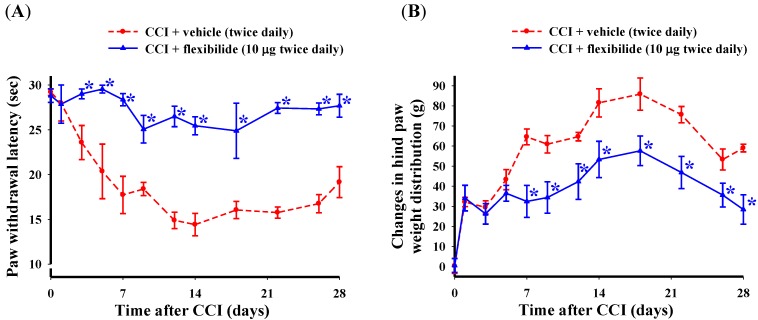
Change in thermal hyperalgesia and weight-bearing deficits after i.t. flexibilide infusion in rats with CCI. (**A**) Time course for the PWL in rats receiving vehicle or flexibilide after CCI; (**B**) Time course of the difference between ipsilateral and contralateral hindpaw weight distribution for rats receiving vehicle or flexibilide after CCI. Within days after CCI, rats developed hypersensitivity to heat stimuli (**A**) and hind paw weight-bearing abnormalities (**B**). Repeated i.t. injection of flexibilide (10 μg twice daily) started on the day of surgery for 28 days almost fully prevented the development of thermal hyperalgesia and weight-bearing deficits (**A**,**B**). Data are shown as mean ± SEM. *n* = 6 per group, * *p* < 0.05 as compared with the CCI + vehicle group at the same time point; CCI: chronic constriction injury, PWL: paw withdrawal latency.

In the next experiment, we evaluated the effect of repeated i.t. flexibilide injections first administered before the CCI injury on the development of thermal hyperalgesia and weight-bearing deficits. Flexibilide or vehicle injection (i.t., 10 μg, twice daily) was initiated on the day of surgery and continued for 28 days. Shortly after CCI, the ipsilateral hindpaw showed a significant lower PWL in response to heat stimulation (Figure 3A) and the difference in weight-bearing between the hind paws also increased (Figure 3B). As compared to vehicle treatment, the repeated i.t. flexibilide treatment significantly prevented development of thermal hyperalgesia starting on day three (Figure 3A) and weight-bearing deficits starting on day seven (Figure 3B) in CCI rats.

### 2.3. Flexibilide Inhibits CCI-Induced Spinal Neuroinflammation

**Figure 4:**
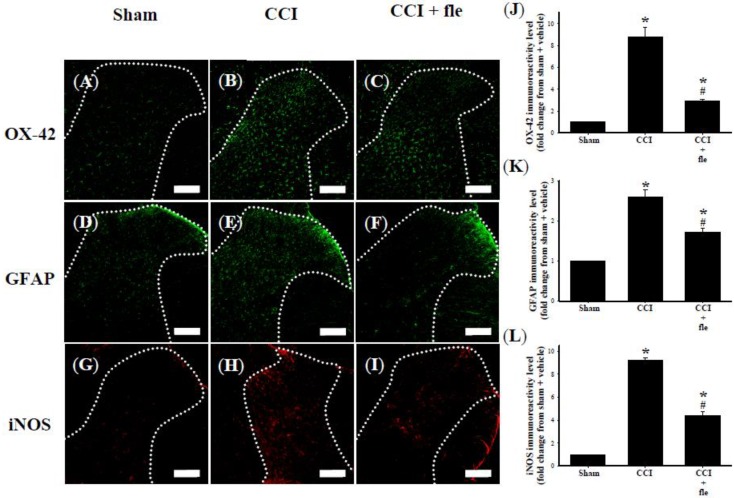
I.t. flexibilide (10 μg twice daily) inhibited the activation of microglial cells and astrocytes as well as the upregulation of iNOS in CCI. Photomicrographs of immunostaining showing microglial cells (green) labeled with OX-42 (a microglial cell-specific marker) (**A**–**C**); astrocytes (green) labeled with GFAP (an astrocyte-specific marker) (**D**–**F**); and expression of iNOS (red) (**G**–**I**) in tissue sections (10 µm) from the sham plus vehicle (**A**,**D**,**G**), CCI plus vehicle (**B**,**E**,**H**), and CCI plus flexibilide (**C**,**F**,**I**) groups. The white dotted line in (**A**–**I**) are indicative of spinal gray matter. Basal levels of the OX-42 (**A**), GFAP (**D**) and iNOS (**G**) signals are observed within the lumbar DH of the sham plus vehicle group. Quantification of OX-42 (**J**), GFAP (**K**), and iNOS (**L**) IR in the lumbar spinal gray matter of the ipsilateral dorsal horn. Spinal immunohistofluorescence indicates a substantial increasein OX-42 (**B**), GFAP (**E**) and iNOS (**H**) IR on the ipsilateral DH at day 14 after CCI surgery. CCI-induced upregulation of OX-42 (**C**), GFAP (**F**) and iNOS (**I**) IR is inhibited by i.t. flexibilide (10 μg twice daily) administered immediately after CCI. Scale bars: 200 µm. *n* = 6 per group, * *p* < 0.05 as compared with the sham + vehicle group; ^#^ *p* < 0.05 as compared with the CCI + vehicle group. i.t.: intrathecal, fle: flexibilide, CCI: chronic constriction injury, iNOS: inducible nitric oxide synthase, GFAP: glial fibrillary acidic protein.

Microglia cells were identified with the OX-42 antibody, which labels cells with the microglial surface marker CD11b. Spinal astrocytes were labeled with an antibody against the glial fibrillary acidic protein (GFAP). OX-42 (Figure 4A) and GFAP (Figure 4D) immunoreactive cells were scattered throughout the ipsilateral DH of the lumbar spinal cord of sham-operated rats. In the ipsilateral spinal DH, both OX-42 (Figure 4B) and GFAP (Figure 4E) immunoreactivity (IR) was clearly increased 14 days after CCI. Significant inhibition of CCI-induced increased OX-42 (Figure 4C) and GFAP (Figure 4F) IR was observed at this time point by i.t. flexibilide administered immediately after surgery and daily for 14 days (10 μg twice per day). Quantification of the OX-42 (Figure 4J) and GFAP (Figure 4K) IR also confirmed that CCI significantly increased the expression of OX-42 and GFAP. Both were significantly inhibited by i.t. flexibilide.

The expression of iNOS was weak in sham-operated rats (Figure 4G). In the CCI rats, iNOS IR was strongly upregulated in the ipsilateral DH of the lumbar spinal cord at the 14th day after CCI (Figure 4H). I.t. flexibilide (10 μg twice daily) immediately after surgery for 14 days prevented this upregulation (Figure 4I). Quantification of iNOS IR demonstrated that i.t. flexibilide significantly suppressed the CCI-induced upregulation of iNOS IR in the spinal DH of CCI rats (Figure 4L). In addition, flexibilide alone did not alter the levels of OX-42, GFAP, or iNOS IR in the spinal dorsal horn in the sham + fle group compared with the sham group.

### 2.4. Flexibilide Attenuates the CCI-Induced Downregulation of TGF-β1

**Figure 5:**
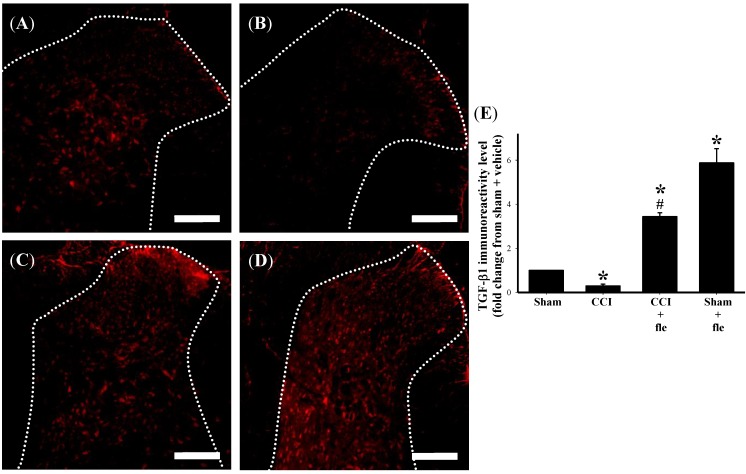
I.t. flexibilide (10 μg twice daily) inhibits the CCI-induced downregulation of TGF-β1 on the DH of the lumbar spinal cord. The white dotted line in (**A**–**D**) are indicative of spinal gray matter. TGF-β1 IR on the ipsilateral DH of the lumbar spinal cord of rats after a sham operation is shown as (**A**); The IR of TGF-β1 on the spinal DH is reduced after CCI (**B**); After i.t. flexibilide injections, the IR of TGF-β1 is increased in the DH of CCI rats (**C**); The IR of TGF-β1 is also increased after i.t. flexibilide infusion in rats with sham surgery (**D**); Quantification of the TGF-β1 IR confirms that TGF-β1 levels are significantly reduced after CCI and that i.t. flexibilide upregulates TGF-β1 expression on the DH of both CCI and sham-operated rats (**E**). Scale bars: 200 µm. *n* = 6 per group, * *p* < 0.05 as compared with the sham + vehicle group; ^#^ *p* < 0.05 as compared with the CCI + vehicle group. CCI: chronic constriction injury, TGF-β1: transforming growth factor-β1, DH: dorsal horn, IR: immunoreactivity.

In order to examine the influence of repeated i.t. flexibilide administration on TGF-β1 expression within the DH of the lumbar spinal cord after CCI, i.t. flexibilide was also injected immediately after CCI for 14 days similar to the above experiments (10 μg, twice daily). We examined the protein expression of TGF-β1 using immunofluorescence. As compared to the sham group (Figure 5A), TGF-β1 IR in the DH of the lumbar spinal cord was clearly reduced at 14 days after CCI surgery (Figure 5B). I.t. flexibilide could prevent the CCI-induced downregulation of TGF-β1 expression in the DH of the lumbar spinal cord (Figure 5C). Interestingly, i.t. flexibilide could even increase the TGF-β1 protein expression in the DH of the lumbar spinal cord in sham-operated rats (Figure 5D). Quantification of the TGF-β1 IR further confirmed that TGF-β1 levels were significantly reduced 14 days after CCI and that i.t. flexibilide significantly increased TGF-β1 expression both in CCI and sham-operated rats (Figure 5E).

**Figure 6:**
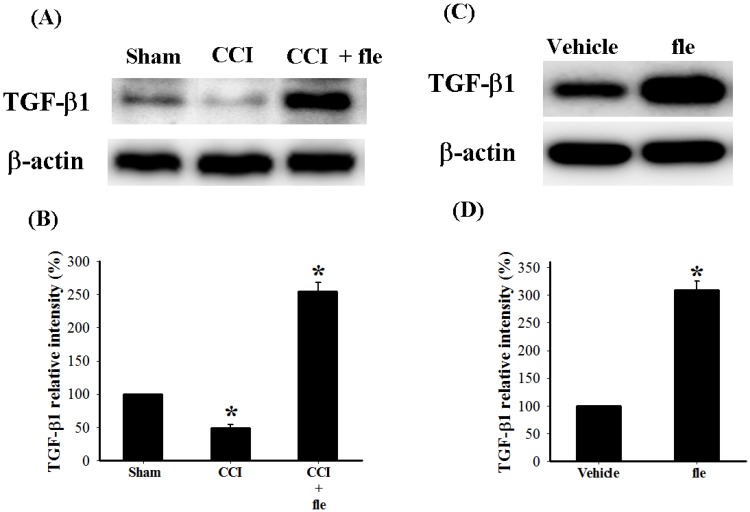
The change in expression of endogenous TGF-β1 protein in the DH of the lumbar spinal cord after CCI and flexibilide injections. (**A**) shows the Western blots for TGF-β1 and β-actin proteins from the spinal DH of rats 14 days after sham surgery, CCI, and CCI plus flexibilide. Quantification of the TGF-β1 protein confirms that TGF-β1 levels are significantly reduced 14 days after CCI and that i.t. flexibilide increases TGF-β1 levels (**B**); (**C**) shows the Western blots for TGF-β1 and β-actin proteins from the spinal DH of sham-operated rats before and after flexibilide infusion. The TGF-β1 levels are increased 30 min after i.t. flexibilide injection. Densitometric quantification of the TGF-β1 protein clearly confirms the findings (**D**). *n* = 3 per group, * *p* < 0.05 as compared with the sham + vehicle group. CCI: chronic constriction injury, TGF-β1: transforming growth factor-β1, DH: dorsal horn.

In order to examine the acute effects of flexibilide on spinal TGF-β1 protein expression in CCI rats, i.t. flexibilide (20 μg) was administered once to rats on the 14th day after CCI. We collected tissues from the ipsilateral lumbar spinal DH from sham rats, CCI rats, and CCI rats injected with flexibilide (30 min after injection) for Western blot analysis. As shown in Figure 6A, TGF-β1 protein expression was significantly decreased 14 days after CCI as compared to the sham group. I.t. flexibilide not only attenuated the CCI-induced TGF-β1 downregulation but also significantly upregulated the TGF-β1 expression (Figure 6A). Quantification of the TGF-β1 IR further confirmed this (Figure 6B). In sham-operated rats, i.t. flexibilide (20 µg) alone significantly upregulated TGF-β1 expression in the ipsilateral lumbar spinal DH (Figure 6C). Quantification of the TGF-β1 IR also confirmed that TGF-β1 levels were significantly increased 30 min after i.t. flexibilide in sham-operated rats (Figure 6D).

### 2.5. The Antinociceptive Effect of Flexibilide is Inhibited by a TGF-β Type I Receptor (TGF-βRI) Inhibitor

**Figure 7:**
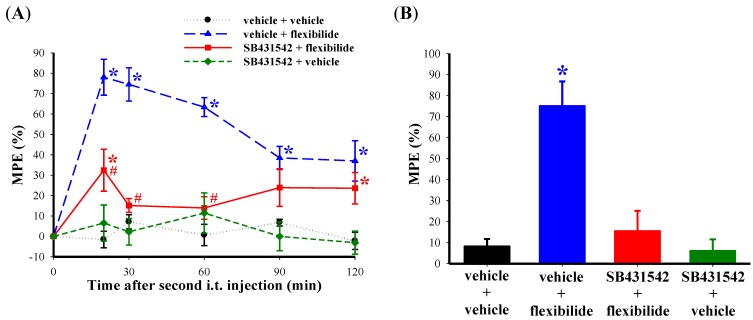
Effect of a TGF-βRI inhibitor on the antinociceptive effects of flexibilide. Fourteen days after CCI, i.t. vehicle or SB431542 (TGF-βRI inhibitor) does not affect CCI-induced thermal hyperalgesia. However, i.t. flexibilide reduces CCI-induced thermal hyperalgesia. The antinociceptive effect of flexibilide is inhibited by pretreatment with SB431542 administered before i.t. flexibilide (**A**). Rats also develop weight-bearing deficits 14 days after CCI. The weight-bearing deficits are reduced in the flexibilide-treated group but not with SB431542 pretreatment (**B**). *n* = 6 per group. * *p* < 0.05 as compared with the vehicle + vehicle group, ^#^ *p* < 0.05 as compared with the vehicle + flexibilide group. CCI: chronic constriction injury. TGF-βRI: TGF-β type I receptor.

In order to confirm the role of TGF-β1 in the analgesic effect of flexibilide, we pretreated rats with a TGF-βRI inhibitor. As expected, i.t. flexibilide at a dose of 20 μg produced a profound inhibition of thermal hyperalgesia (Figure 7A) and weight-bearing deficits (Figure 7B). I.t. administration of the selective TGF-βRI inhibitor SB431542 (2 μg) one hour prior to flexibilide injection in CCI rats attenuated the effects of flexibilide in both thermal hyperalgesia (Figure 7A) and in the weight-bearing deficits (Figure 7B). I.t SB431542 (2 μg) when administered alone did not change the thermal hyperalgesia (Figure 7A) and weight-bearing deficits in CCI rats (Figure 7B).

## 3. Discussion

### 3.1. Summary

The purpose of this study was to examine the anti-inflammatory properties of flexibilide and its role in attenuating nociception and spinal neuroinflammation in CCI rats. We also evaluated the role of spinal TGF-β1 in the antinociceptive effect of flexibilide in CCI rats. Our data support the hypothesis that flexibilide may serve as an analgesic compound in neuropathic pain. First, i.t. flexibilide significantly inhibited the established thermal hyperalgesia in CCI rats and that repeated i.t. flexibilide administration immediately after CCI could prevent development of thermal hyperalgesia and weight-bearing deficits. Second, flexibilide inhibited the CCI-induced activation of spinal microglia and astrocytes. Third, flexibilide markedly inhibited the expression of the spinal proinflammatory mediator iNOS. Fourth, flexibilide inhibited the CCI-induced downregulation of spinal TGF-β1. Fifth, the antinociceptive effect of flexibilide was inhibited by a TGF-βRI inhibitor. These results suggest that flexibilide has the capacity to attenuate nociceptive sensitization by modulating spinal neuroinflammatory processes and TGF-β1 in peripheral neuropathy.

### 3.2. Analgesic Effects of i.t. Flexibilide in Neuropathic Pain

Neuropathic pain is defined as pain caused by a dysfunction or damage to the nervous system including peripheral nerves and certain CNS regions [48,49]. It is characterized by spontaneous pain, allodynia (pain in response to normally innocuous stimuli), and hyperalgesia (aggravated pain in response to noxious stimuli) [50]. However, direct evaluation of spontaneous pain in rats is not possible. Instead, indirect signs of weight-bearing deficits are often considered indications for the presence of spontaneous pain [51]. In addition, thermal hyperalgesia, as measured as the PWL to radiant heat, is indicative of evoked pain [52] in our study. We chose the CCI animal model because behavioral changes relevant to spontaneous pain are particularly prominent in the CCI model as compared to the partial sciatic nerve ligation (PSNL) and spinal nerve ligation (SNL) models [52]. Therefore, analgesic effects for flexibilide could be more closely relate to clinically relevant effects on spontaneous pain. In this study, we did indeed find that i.t. flexibilide alleviates thermal hyperalgesia in rats when administered on the 14th day after CCI. In addition, i.t. flexibilide twice a day immediately after CCI reduced thermal hyperalgesia and weight-bearing deficit in rats over a 28-day period. These results suggest that spinally administered flexibilide could be an effective analgesic agent for thermal hyperalgesia and could reduce gait abnormalities in neuropathic pain.

### 3.3. The Effect of Flexibilide on Glial Cell Activation

Neuroinflammation in neuropathological conditions is characterized by activation of microglia and astrocytes [12,14,15,53]. Both activated microglia and astrocytes release pro-inflammatory cytokines, which can cause spinal nociceptive sensitization in neuropathic pain [12,54,55]. Several studies using immunohistochemistry have also demonstrated that the activation of astrocytes and microglia parallels nociceptive behaviors following peripheral nerve injury [55,56]. Many studies suggest that microglia are involved in the initiation of neuropathic pain, whereas astrocytes function to maintain neuropathic pain [20,21,57,58]. This is also demonstrated pharmacologically as inhibition of microglia attenuates development of neuropathic pain, whereas inhibition of astrocytes attenuates existing behavioral hypersensitivity in neuropathy [20,27]. Both minocycline, which selectively disrupts the activation of microglia, and fluorocitrate, which disrupts astrocyte activation, can inhibit neuropathic symptoms such as allodynia and hyperalgesia in animal models [20,23,24,59]. As with previous studies which inhibit glial cell activation, our data shows that flexibilide could prevent both the development of thermal hyperalgesia and attenuate existing thermal hyperalgesia in CCI rats. This was associated with a significant inhibition of spinal microglial and astrocyte activation. The present results using flexibilide provide further evidence that pharmacological disruption of glial cell activation can reduce neuroinflammation and promote antinociception in neuropathic pain states.

### 3.4. The Role of TGF-β in the Spinal Neuroinflammation Induced Neuropathic Pain

The TGF-β family belongs to a superfamily of multifunctional cytokines with important functions in the modulation of cell proliferation, differentiation, apoptosis, adhesion, migration, and in extracellular matrix production [60]. TGF-β family members can initiate intracellular signaling by inducing the assembly of a heterotetrameric complex comprised of the TGF-β type I and type II transmembrane receptors (TGF-βRI and TGF-βRII) [61,62]. Upon ligand binding to TGF-βRII, the TGF-βRI can recognize TGF-βRII and be subsequently recruited into the receptor signaling complex [60]. TGF-βRII phosphorylates TGF-βRI in a region rich in glycine and serine/threonine residues inducing a conformational change of TGF-βRI and activating TGF-βRI kinases [63]. Type I receptors then phosphorylate Smad proteins, which translocate into the nucleus to regulate gene transcription and induce downstream signaling pathways [60].

Increasing evidence has shown TGF-β to be a particularly potent anti-inflammatory cytokine. Several studies demonstrate anti-inflammatory effects in LPS-stimulated macrophage cells [64,65,66,67]. Both exogenous and endogenous TGF-β1 can suppress activation and proliferation of microglia and astrocytes [40,42,68] thereby exerting both anti-inflammatory and neuroprotective actions [41]. These actions have been implicated in various neuropathological disorders [36,69,70,71,72]. Further, several studies have indicated that TGF-β1 can inhibit microgliosis and activation of microglia and astrocytes in the spinal cord of rats with peripheral neuropathy [43,44]. A previous study from our group also showed that i.t. administration of TGF-β1 reduced the expression of the spinal pro-inflammatory protein TNF-α in CCI rats [44]. All these findings indicate that TGF-β1 reduces neuroinflammation by suppressing glial cell activation and thereby reducing pro-inflammatory cytokine release.

### 3.5. The Impact of TGF-β on the Analgesic Effects of Flexibilide

Flexibilide was shown to possess anti-inflammatory activities since it was capable to reduce carrageenan-induced paw edema [47]. In a previous *in vitro* study from our group, we found that flexibilide could significantly inhibit upregulation of inducible nitric oxide synthase (iNOS) and cyclooxygenase-2 (COX-2), as well as upregulated TGF-β1 in lipopolysaccharide (LPS)-stimulated murine macrophage RAW 264.7 cells [6]. In addition, flexibilide was able to upregulate production of TGF-β1 in carrageenan-induced inflamed paw tissue of rats [6]. In the current study, as with previous studies, i.t. flexibilide not only prevented CCI-induced downregulation of spinal TGF-β1, but even increased above basal levels.

TGF-β1 has been found to have antinociceptive effects in previous studies. For example, i.t. infusion of recombinant TGF-β1 was shown to attenuate the development of thermal hyperalgesia and mechanical allodynia, as well as reverse established nociception in rats with peripheral nerve injury [43,44]. To examine the role of TGF-β1 in the anti-nociceptive effects of flexibilide, we administered the TGF-βRI inhibitor, SB431542, prior to i.t. flexibilide in CCI rats. To estimate an effective i.t. dosage of SB431542, we calculated the *in vivo* dosage for central administration based on the *in vitro* dosage with a previous formula developed by Caraci *et al.* [73], considering that the molecular weight of SB431542 is 384.39 and the CSF volume of a 300 g rat equals about 580 µL [74]. Hence, we estimated that the dosage of SB431542 for i.t. administration to a rat was approximately 2 µg, which reaches a concentration of about 10 µM of SB431542 in the CSF. This concentration exceeds the IC_50_ value of SB431542 for the TGF-β1 receptor by about 100 fold [75]. This selected dosage was administered 1 h before flexibilide injection, for subsequent tests. The antinociceptive effect of flexibilide was significantly attenuated by the TGF-βRI inhibitor (Figure 7A,B). Based on our observations and those of others, we propose that TGF-β1 plays an important role in the antinociceptive effects of flexibilide.

### 3.6. The Relationship between Flexibilide, TGF-β1 and iNOS

Reactive oxygen species such as NO and superoxide exert multiple important modulatory effects on inflammatory and immune responses [76,77]. They are released by neutrophils [78], astrocytes [79] and microglia [80,81,82] under inflammatory conditions. NO is a diffusible free radical which is synthesized by NOS. There are three different forms of NOS, of which the neuronal and endothelial forms (nNOS and eNOS) are constitutive, while the iNOS is upregulated in immune cells such as macrophages and glial cells under inflammatory states [83]. When produced in excess, NO reacts with superoxide radicals to form peroxynitrite, a powerful oxidant that can cause tissue damage. Several reports have suggested that high levels of NO are involved in the development and maintenance of neuropathic pain [31,84]. NO is also implicated in the development and maintenance of central nociceptive sensitization after peripheral tissue damage or inflammation [85]. Systemic or i.t. administration of nitroglycerin, an NO donor, induces hyperalgesia in rats [32,86,87]. Further, i.t. delivery of the non-specific NOS inhibitor L-N^G^-nitroarginine methyl ester (L-NAME) produces a dose-dependent reduction of thermal hyperalgesia in CCI rats [88].

Many studies have shown that activated microglial cells and astrocytes produce NO via induction of iNOS [82,89]. In nociceptive conditions, inhibition of glial cell activation with the glial cell inhibitor, fluorocitrate, significantly inhibits the upregulated NOS expression and activity, and consequently NO production [90]. Consistent with previous studies on carbon monoxide [33] and fluorocitrate [90], we found that i.t. flexibilide inhibited glial cell activation and reduced the CCI-induced upregulation of spinal iNOS. TGF-β1/β2 is known to suppress NO production in co-cultures of rat microglial and astroglial cells after stimulation of the cells with lipopolysaccharide [81]. In addition, both aberrant expression of iNOS and increased NO production have been noted in TGF-β1 knockout mice [91]. This inverse relationship between upregulation of TGF-β1 and downregulation of iNOS after i.t. flexibilide was also observed in our study. Since TGF-β1 has been shown to inhibit activation of microglia and astrocytes in previous studies [41,43,44], the suppressive effect of i.t. flexibilide on iNOS is probably secondary to the up-regulation of TGF-β1. Therefore, we can now speculate that flexibilide inhibits glial cell activation and iNOS expression through up-regulation of TGF-β1.

### 3.7. Possible Mechanism of Flexibilide in Treating Neuropathic Pain

**Figure 8:**
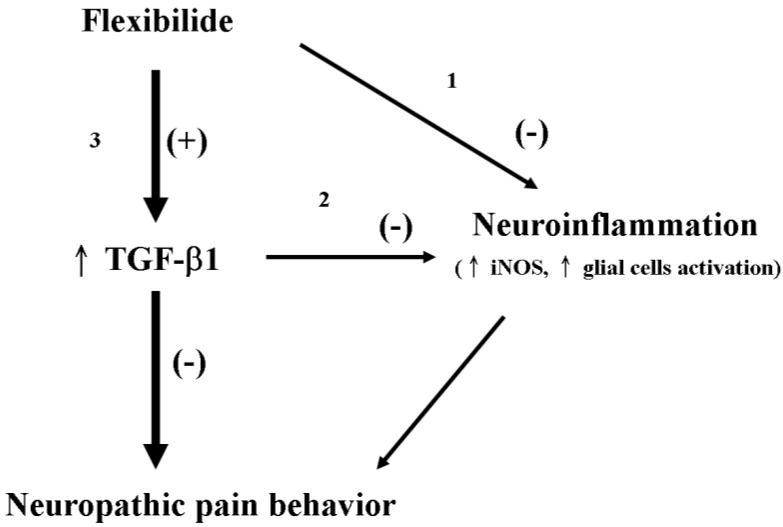
Schematic representation of the possible mechanisms by which flexibilide reduces neuropathic pain. Flexibilide reduces neuropathic pain by directly inhibiting the expression of iNOS and the activation (+) of microglia and astrocytes (pathway 1). Through upregulation of TGF-β1 (↑) by flexibilide, the expression of iNOS and the activation of microglia and astrocytes are inhibited (−) (pathway 2). Flexibilide may also upregulate TGF-β1 to attenuate neuropathic pain behavior directly (pathway 3). TGF-β1: transforming growth factor-β1, iNOS: inducible nitric oxide synthase.

The antinociceptive effects of flexibilide can be speculated to occur in three different manners. First, flexibilide may directly inhibit upregulation of iNOS and activation of glial cells to reduce neuroinflamation, which may improve neuropathic pain behavior in CCI rats. Second, flexibilide may indirectly inhibit upregulation of iNOS and glial cell activation through up-regulation of TGF-β1 to alleviate neuropathic pain. Third, flexibilide can directly upregulate TGF-β1, which itself shows antinociceptive effects. Since the antinociceptive effects of flexibilide were significantly reduced by administration of the TGF-βRI inhibitor, we propose that upregulation of TGF-β1 by flexibilide must play an important role in the antinociceptive effects of flexibilide (Figure 8).

### 3.8. The Advantages of Flexibilide Obtained from Cultivated Soft Coral

More than 50% of currently approved drugs were originally discovered in natural sources. Therefore, natural compounds remain an integral source for the development and discovery of drugs. As the natural compounds present in marine organisms often have a high complexity and specific bioactivity, they can possess considerably different characteristics from those found in terrestrial organisms. They can hence be crucial for research purposes and drug development in medicine [10,92]. However, a critical problem of drug development from new molecules originating from natural sources is a sustainable supply of compounds, which are normally present only in low amounts and/or can be very difficult to isolate or synthesize. In the current study, flexibilide was isolated from the soft coral *Sinularia flexibilis*, which was cultured in a culture tank of the National Museum of Marine Biology and Aquarium in Taiwan [93]. Such a constant supply of cultivated soft corals *Sinularia flexibilis* provides a great advantage to perform preclinical and clinical trials without a supply problem.

## 4. Methods and Materials

### 4.1. Preparation of Flexibilide

In this study, flexibilide was isolated and purified from soft coral, *Sinularia flexibilis*, cultured in a culture tank of the National Museum of Marine Biology and Aquarium in Taiwan. The method of flexibilide extraction was modified from that of Lu *et al.* [94,95]. The specimens were extracted with 95% ethanol. The crude extract was further partitioned between *n*-hexanes (*n*-hexanes: water = 1:1) and ethyl acetate (ethyl acetate: water = 1:1). The *n*-hexane-ethyl acetate layer was separated over normal phase silica gel (silica gel 60, 230–400 mesh, Silicycle, QC, Canada) by column chromatography and eluted with *n*-hexane, ethyl acetate, acetone, and methanol to yield 29 fractions. Fraction 12 was eluted with *n*-hexanes and ethyl acetate (1:1) over normal phase silica gel to generate flexibilide. The structure of flexibilide was identified by nuclear magnetic resonance spectroscopy (NMR) [45]. The purity (>98%) of flexibilide was identified and verified by ^1^H-NMR and ^13^C-NMR spectra (Varian Mercury Plus 400 FT-NMR at 400 MHz, Varian, CA, USA) (Supplementary Figures S1–S3).

### 4.2. Animals

Male Wistar rats (260–285 g body weight; BioLASCO Taiwan Co., Ltd., Taipei, Taiwan) were maintained in a temperature-controlled (22 ± 1 °C) room with 12-h light/dark cycle. Food and water were provided ad libitum. Surgery and drug injections were performed on all rats under isoflurane (2%)-inhaled anesthesia. To prevent infection, rats received a postoperative injection of Veterin (cefazolin; 0.17 g/kg) intramuscularly. All animal experiments were approved by the National Sun Yat-sen University Animal Care and Use Committee and complied with the Guiding Principles in the Care and Use of Animals of the American Physiology Society. Every effort was taken to minimize animal suffering and the number of animals used.

### 4.3. I.t. Catheter Implantation and Induction of Peripheral Neuropathy

For spinal injection of flexibilide or vehicle, the rats were implanted with i.t. catheters (polyethylene tubes [PE5]: 9 cm, 0.008-inch inner diameter, 0.014-inch outer diameter; Spectranetics, Colorado Springs, CO, USA). According to the method described by Yaksh and Rudy [96] and from our previous study [25], we threaded the i.t. catheter to the rat’s lumbar enlargement of the spinal cord by inserting it through the cisternal membrane at the base of the skull. The end of the i.t. catheter was externalized and fixed to the cranial aspect of the rat’s head for spinal injection. At 5 days after this surgery, rats that displayed fresh blood in the cerebrospinal fluid (CSF) or evidence of gross neurological injury were excluded from subsequent experimentation and sacrificed. After the 5-day recovery period, rats received CCI surgery to the right sciatic nerve in accordance to the method first described by Bennett and Xie [97] and used previously in our studies [26,98]. After exposure of the right sciatic nerve at the mid-thigh level, a 5 mm long nerve segment was isolated and four loose ligatures (4–0 chromic gut) were placed around the sciatic nerve with 1-mm interval spacing between them. The muscle and skin incisions were then closed with sutures. Sham-operated rats received the same surgery and exposure of the right sciatic nerve but with no ligation. We evaluated the locomotor function of rats with the BBB locomotor scale using scores ranging from 0 (complete paralysis) to 21 (normal locomotion) [99] according methods previously described [100] and our previous studies [25,26]. After placing rats into transparent Plexiglas boxes, two observers scored hind limb movements and walking behaviors for 4 min. Scores between 0 and 7 suggest minimal movement of individual hind limb joints (hip, knee, and ankle); scores between 8 and 13 suggest abnormal gait (paw placement and stepping) and disturbed forelimb–hindlimb coordination; and scores between 14 and 21 reflect toe clearance, predominant paw position, trunk stability, and ability to keep the tail raised.

### 4.4. Effects of i.t. TGF-β1 Injection on CCI-Induced Gait Abnormalities and Nociception

In order to evaluate the potential effects of flexibilide on CCI-induced weight-bearing deficits and thermal hyperalgesia, we designed three sets of experiments:

(1) To measure the antinociceptive effects of different doses of flexibilide on CCI-induced neuropathy, the following six treatments were administered at 14 days post-CCI:
  - i.t. vehicle
  - i.t. flexibilide (1 μg)
  - i.t. flexibilide (5 μg)
  - i.t. flexibilide (10 μg)
  - i.t. flexibilide (20 μg)
  - i.t. flexibilide (50 μg)
(2) In order to explore the effect of preventative i.t. flexibilide treatment on the development of neuropathic pain, the following two treatment groups were used:
  - i.t. vehicle (10 μg, twice daily for 4 weeks immediately after CCI surgery)
  - i.t. flexibilide (10 μg, twice daily for 4 weeks immediately after CCI surgery)

We chose the above treatment protocol based on two considerations. First, i.t. 20-μg flexibilide was able to maintain an antinociceptive effect of over 50% MPE for more than 80 min after CCI (Figure 2A). Second, dividing the 20-μg flexibilide dose in half and administering it twice daily could prolong the antinociceptive effect and reduce potential cumulative toxicity over the 28-day time course.

(3) In order to explore the effect of SB431542 (catalog no. S4317; Sigma Co., Ltd., St Louis, MO, USA), a selective inhibitor of the TGF-βRI [73], on the antinociceptive effects of flexibilide in CCI-induced neuropathy, the following four groups of rats were used at 14 days post-CCI:
  - i.t. vehicle + vehicle (60 min after the first injection);
  - i.t. vehicle + flexibilide (20 μg)
  - i.t. SB431542 (2 μg) + flexibilide (20 μg)
  - i.t. SB431542 (2 μg) + vehicle

Flexibilide or vehicle was delivered in a volume of 10 μL artificial CSF (aCSF), consisting of 122.7 mM Cl^−^, 21.0 mM HCO_3_^−^, 2.5 mM HPO_4_^2−^, 151.1 mM Na^+^, 0.9 mM Mg^2+^, 1.3 mM Ca^2+^, 3.5 mM dextrose, and 2.6 mM K^+^. The final pH was adjusted to 7.3 by bubbling with oxygen containing 5% CO_2_. To ensure complete drug delivery, all i.t. catheters were flushed with 10 μL aCSF to take into account the 3.5-μL dead volume of the i.t. catheter.

Thermal hyperalgesia was assessed using an analgesiometer (IITC Inc., Woodland Hills, CA, USA) that measures the PWL, as described by Hargreaves *et al.* [101] and in our previous study [6]. We placed rats in clear plastic boxes on the top of an elevated glass plate and targeted the plantar surface of the ipsilateral hind paw using a low-intensity radiant heat source (arbitrary intensity setting = 25). A positive response (licking or withdrawal of hind paw after heat stimulus) indicative of a pain behavior was measured manually using a digital timer. A cutoff time of 30 s was used to prevent tissue damage.

To evaluate weight-bearing deficits, we placed rats on an incapacitance tester (Singa Technology Corporation, Taipei, Taiwan) with their hindpaws centered on two force transducers, which measured the weight distribution between the hind limbs using a method described in our previous study [102]. Under normal conditions, naïve rats distribute their weight equally on both hind limbs. However, after peripheral inflammation or nerve injury, rats reduce their weight on the affected limb [103]. We expressed the change in hindpaw weight distribution in grams as the difference between the ipsilateral hindlimb and the contralateral hindlimb measured at the same time point. 

In the first and third experimental sets (see above), we transformed PWL data (s) and changes in hind paw weight distribution (g) of rats to the percentage of the MPE using the following formula: %MPE = (post-drug latency − baseline)/(cut off − baseline) × 100; baseline: measurement immediately before i.t. injection of flexibilide or vehicle, post-drug latency: measurement after i.t. injection of flexibilide or vehicle, cut off: cutoff time of 30 s or cutoff weight of 0 g. In the first set of rats, in order to simplify data analysis, the area under the curve (AUC) for the plot of %MPE *versus* time from each rat was calculated with the trapezoidal method [104], from 0 to 120 min after i.t. injection.

### 4.5. Spinal Immunohistofluorescence

Using a spinal immunohistofluorescence method previously described in our studies [6,26], we collected the lumbar enlargement (L2–L4) of spinal tissue from the following 4 groups of rats 30 min after vehicle or flexibilide treatment and at 14 days after CCI or sham surgery:

(1) sham-operated plus i.t. vehicle (administered twice daily for 2 consecutive weeks immediately after sham surgery)
(2) CCI plus i.t. vehicle (administered twice daily for 2 consecutive weeks immediately after CCI surgery)
(3) CCI plus i.t. flexibilide (administered twice daily for 2 consecutive weeks immediately after CCI surgery)
(4) sham-operated plus i.t. flexibilide (administered twice daily for 2 consecutive weeks immediately after sham operated).

We mounted the lumbar spinal cord tissues from the different groups together in the same OCT block in order to decrease the variability of the immunohistochemical procedures [25,105]. After sectioning the OCT block with a cryostat at −30 °C (HM550; Microm International GmbH, Walldorf, Germany), the spinal sections (at a thickness of 10 μm) were incubated overnight at 4 °C with the following primary antibodies: anti-OX-42 (CD11b, microglia marker, 1:200 dilution, cat. CBL1512; EMD Millipore, Temecula, CA, USA), GFAP (astrocyte marker, 1:200 dilution, cat. 131-17719; Life Technologies Corporation, Grand Island, NY, USA), iNOS (1:200 dilution, cat. 6103322; BD Pharmingen, San Diego, CA, USA), or anti-TGF-β1 (1:200 dilution, cat. ab92486; Abcam, Cambridge, UK). This was then followed by incubation for 40 min at room temperature with the following secondary antibodies: Alexa Fluor 488-labeled chicken anti-mouse IgG antibody (1:400 dilution; Life Technologies Corporation; catalog No. A-21200; green fluorescence) or DyLight 549-conjugated donkey anti-rabbit IgG antibody (1:400 dilution; Jackson ImmunoResearch Laboratories, Inc., West Grove, PA, USA; catalog No. 711-506-152; red fluorescence). We examined the spinal tissue sections using a Leica DM-6000 CS fluorescence microscope (Leica Microsystems GmbH, Wetzlar, Germany), acquired images using a SPOT Xplorer Digital camera (Diagnostic Instruments, Inc., Sterling Heights, MI, USA), and then counted the pixel values of the positive areas with the MetaVue Imaging software (Molecular Devices LLC, Downingtown, PA, USA). For quantification of IR, we focused on laminae I–III of the lumbar spinal gray matter in the ipsilateral dorsal horn of the rats. The immunohistochemical images of OX-42, GFAP, iNOS, and TGF-β1 for quantification were taken at 100× magnification and under the same exposure conditions, respectively. Immunohistochemical data (average intensity of fluorescence per pixel, AIFP) were represented as fold change as compared to the sham-operated and vehicle group, which were normalized as a fold change of 1.

### 4.6. Spinal Western Blot Analysis

We executed Western blot analysis on ipsilateral dorsal side of the lumbar spinal cord from rats using a method previously used in our studies [106,107]. The following five experimental groups were used at 14 days after CCI or sham surgery:

(1) sham-operated plus i.t. vehicle
(2) CCI plus i.t. vehicle
(3) CCI plus i.t. flexibilide
(4) sham-operated plus i.t. vehicle
(5) sham-operated plus i.t. flexibilide

We homogenized the spinal samples in ice-cold lysis buffer (pH 7.5, 50 mM Tris, 100 μg/mL phenylmethylsulfonyl fluoride, 1% Triton X-100, 1 μg/mL aprotinin, 150 mM NaCl) with a Polytron homogenizer (5 cycles of 10 s at 3000 rpm). The supernatant was collected after centrifuging at 20,000× *g* for 60 min at 4 °C. Protein determination of the supernatant was performed using the DC protein assay kit (Bio-Rad, Hercules, CA, USA) according to the modified method of Lowry *et al.* [108]. After the addition of an equal volume of sample buffer (50 mM Tris–HCl, pH 7.2, 2% 2-mercaptoethanol, 2% sodium dodecyl sulfate (SDS), 0.1% bromophenol blue, and 10% glycerol) into the supernatant, we electrophoresed the proteins in the supernatant using a tricine SDS-polyacrylamide gel with 150 V for 90 min. Subsequently, we transferred the proteins within the gel into a polyvinylidene difluoride membrane (PVDF membrane; Immobilon-P, Millipore, 0.45-μM pore size) in transfer buffer (1% SDS, 50 mM Tris–HCl, 380 mM glycine, 20% methanol) with 125 mA overnight at 4 °C. After blocking the PVDF membrane with 5% non-fat dry milk in Tris-buffered saline (TTBS; 0.1% Tween 20, 137 mM NaCl, 20 mM Tris-HCl, pH 7.4) for 1 h at room temperature, the PVDF membrane was incubated with antibody against TGF-β1 (1:1000 dilution) protein for 180 min at room temperature. The antibody recognized the immunoreactive band of TGF-β1 protein (~44 kDa) which was visualized using enhanced chemiluminescence (ECL kit; Millipore) and then photographed with the UVP BioChemi imaging system (UVP, LLC, Upland, CA, USA). Finally, we performed relative densitometric quantification of the immunoreactive bands of TGF-β1 protein using LabWorks 4.0 software (UVP, LLC, Upland, CA, USA). Differences between the bands in the different groups were calculated within the same image. In addition, we reprobed the PVDF membranes with an anti-β-actin antibody (1:2500 dilution; catalog no. A5441; Sigma Co., Ltd., St Louis, MO, USA; monoclonal mouse antibody), which was used as a loading control.

### 4.7. Data and Statistical Analyses

All data are presented as means ± standard error of the mean (SEM). We analyzed the differences between the groups using a one-way analysis of variance (ANOVA) and the Student–Newman–Keuls *post hoc* test. Statistical significance was recognized when *p* values were less than 0.05.

## 5. Conclusions

The antinociceptive effects of flexibilide in the CCI model of neuropathic pain have not been previously reported. The present study demonstrates that i.t. flexibilide can reduce CCI-induced thermal hyperalgesia and weight-bearing deficits in rats. Suppression of glial cell activation and downregulation of iNOS by flexibilide is accompanied with upregulation of TGF-β1 expression in the dorsal horn of the spinal cord, suggesting the involvement of TGF-β1 in the anti-neuroinflammatory and analgesic effects of flexibilide.

## Supplementary Files

Supplementary File 1 Supplementary Information (PDF, 350 KB)
