# Supplementary material for: Flexibilide Obtained from Cultured Soft Coral Has Anti-Neuroinflammatory and Analgesic Effects through the Upregulation of Spinal Transforming Growth Factor-β1 in Neuropathic Rats
Source: Mar Drugs. 2014 Jun 27;12(7):3792–817. doi: 10.3390/md12073792 (PMC4113799; doi:10.3390/md12073792)
Supplement: Supplementary File 1 — Supplementary Information (PDF, 350 KB) [file marinedrugs-12-03792-s001.pdf]

Supplementary Information

Figure S1. ^{1}H-NMR spectrum of flexibilide in $CDCl_3$ at 400 MHz.

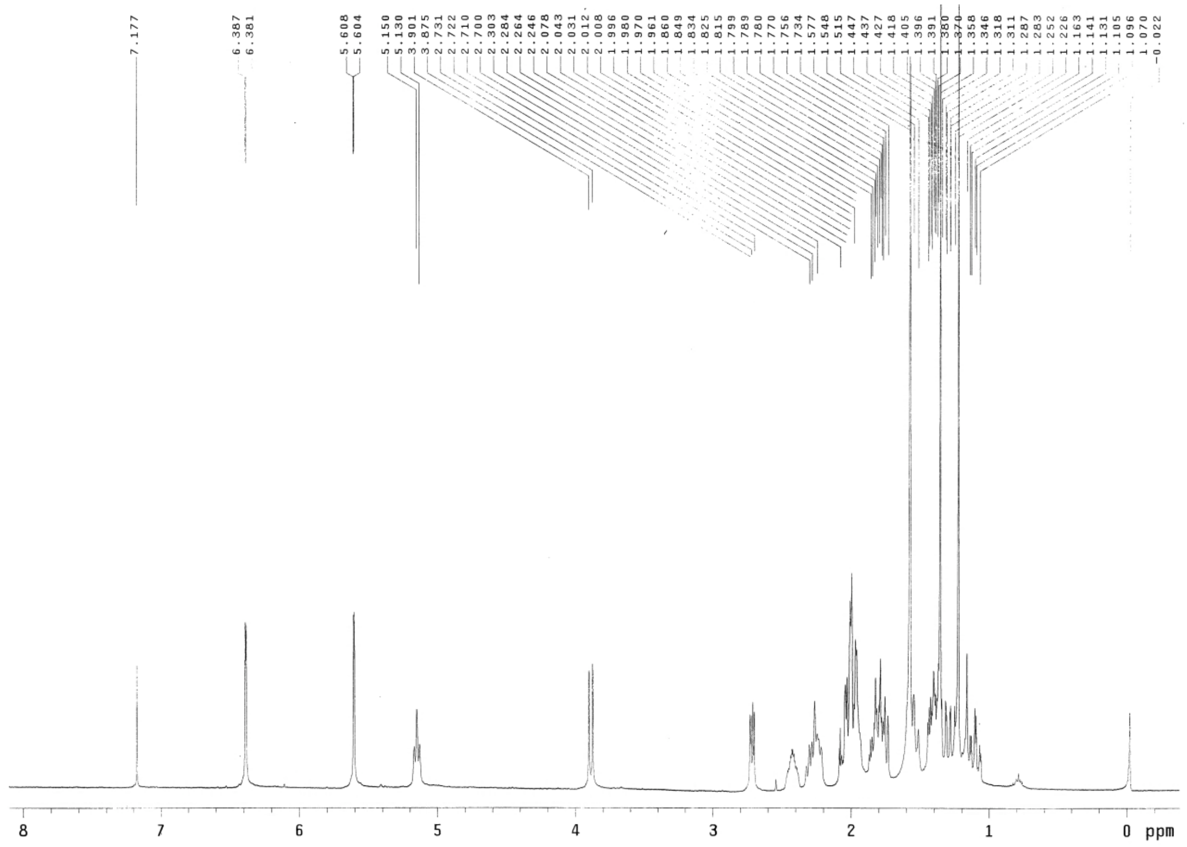

Figure S2. ^{13}C-NMR spectrum of flexibilide in $CDCl_3$ at 100 MHz.

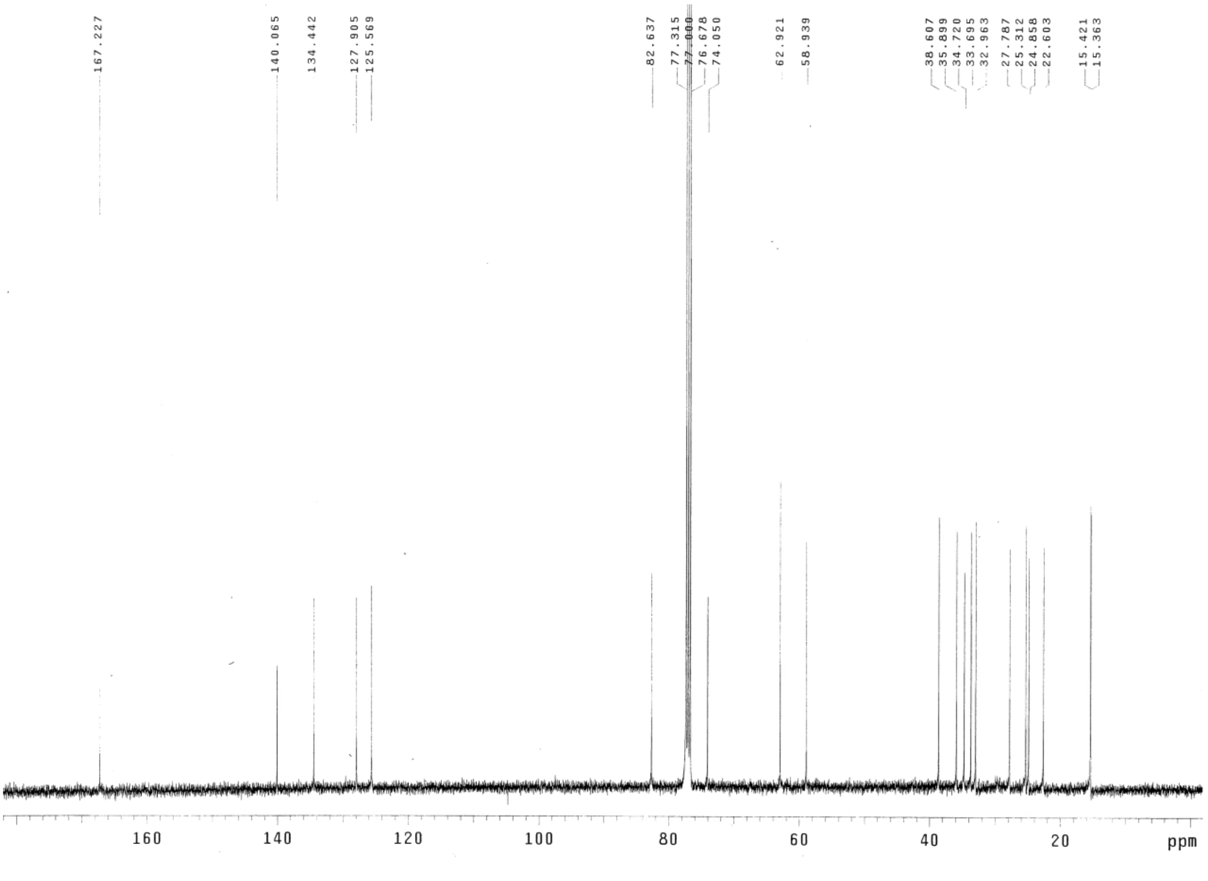

Figure S3. ESIMS spectrum (*m/z* 357 [M + Na]$^+$) of flexibilide.

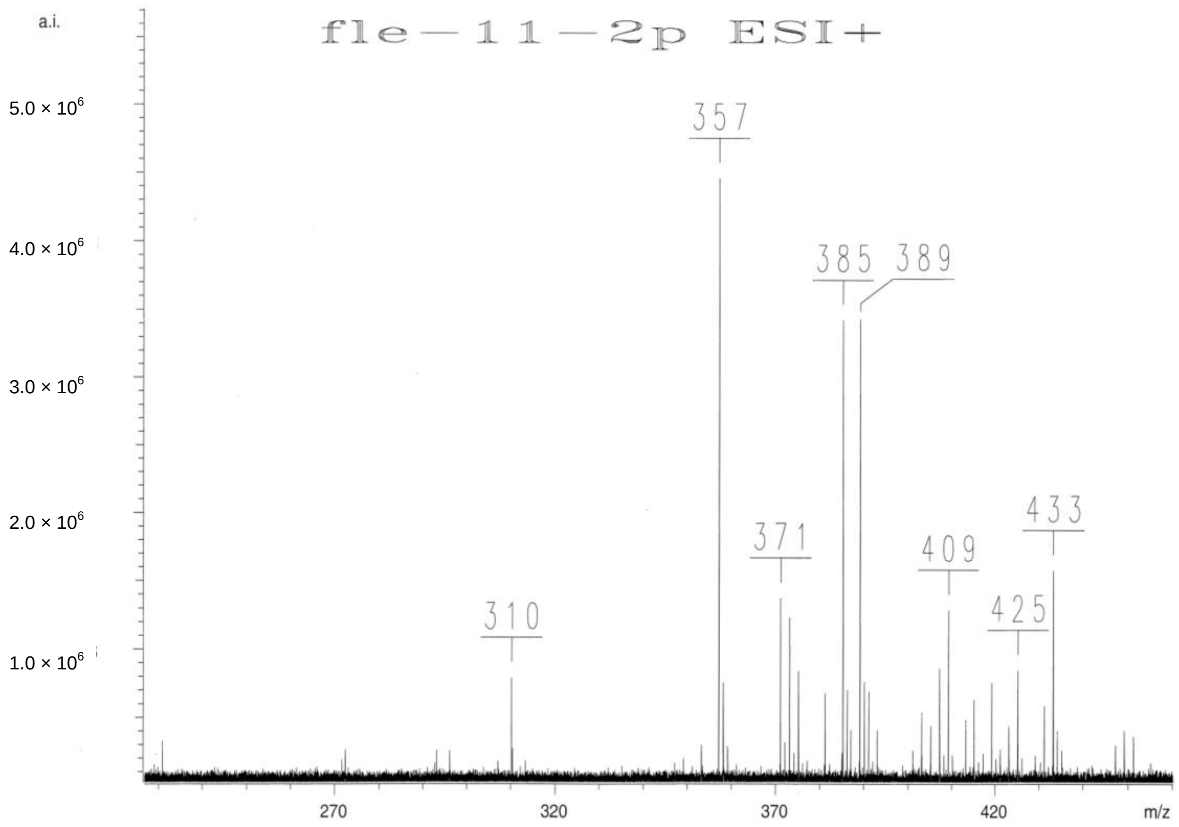

© 2014 by the authors; licensee MDPI, Basel, Switzerland. This article is an open access article distributed under the terms and conditions of the Creative Commons Attribution license (http://creativecommons.org/licenses/by/3.0/).
